# Highly Pathogenic H5N1 Influenza Virus in Smuggled Thai Eagles, Belgium

**DOI:** 10.3201/eid1105.050211

**Published:** 2005-05

**Authors:** Steven Van Borm, Isabelle Thomas, Germaine Hanquet, Bénédicte Lambrecht, Marc Boschmans, Gérald Dupont, Mireille Decaestecker, René Snacken, Thierry van den Berg

**Affiliations:** * Veterinary and Agrochemical Research Centre, Brussels, Belgium;; † Scientific Institute of Public Health, Brussels, Belgium

**Keywords:** Influenza A, Europe, H5N1

## Abstract

We report the isolation and characterization of a highly pathogenic avian influenza A/H5N1 virus from Crested Hawk-Eagles smuggled into Europe by air travel. A screening performed in human and avian contacts indicated no dissemination occurred. Illegal movements of birds are a major threat for the introduction of highly pathogenic avian influenza.

The 2003–2004 highly pathogenic avian influenza epidemic caused by an A/H5N1 virus has become established in 8 Southeast Asian countries (1). It affects not only birds but several mammals (2–4). As of February 2005, 55 laboratory-confirmed human cases, 42 fatal, have occurred after direct transmission of the virus (available from http://www.who.int/csr/disease/avian_influenza/country/en/).

## The Study

On October 18, 2004, 2 Crested Hawk-Eagles (*Spizaetus nipalensis*) smuggled into Europe from Thailand were seized at Brussels International Airport (5). Clinical examination of the birds showed no symptoms. As import of birds and products from several Asian countries into the European Union (EU) is forbidden (DG Sanco Decision 2004/122/EC), the birds were humanely sacrificed and immediately sent to the Veterinary and Agrochemical Research Centre for routine diagnosis to exclude influenza and Newcastle disease viruses.

**Figure 1:**
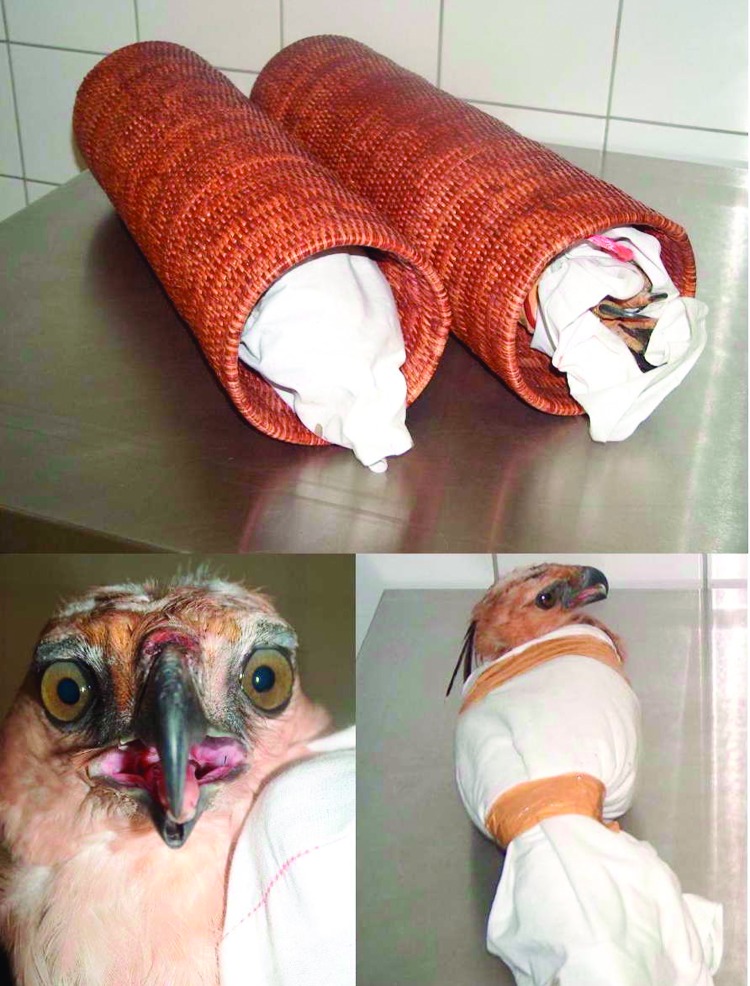
Crested Hawk-Eagles confiscated at Brussels International Airport in the hand luggage of a Thai passenger. The birds were wrapped in a cotton cloth, with the heads free, and each of them inserted in a wicker tube ≈60 cm in length, with 1 end open. Pictures courtesy of Paul Meuleneire, custom investigations officer, antidrug group.

The eagles were transported in a hand luggage (Figure 1), with the zipper not totally closed to allow air to enter. The bird smuggler, a Thai resident, took connecting flights from Bangkok to Brussels, with a stopover in Vienna; he placed his hand luggage in an overhead compartment during both flights. When interviewed, the Thai man declared that he "bought the eagles on a major Bangkok market, a few days before departure, as a present for [his] brother living in Belgium." The eagles reportedly had no contact with domestic animals before departure. A few days later, a Belgian falconer declared he had ordered the eagles and offered €7,500 for each bird.

Necropsy and further testing were carried out at the Veterinary and Agrochemical Research Centre (virologic procedures according to EU Council Directive 92/40/EEC of 19 May 1992). Both eagles had enteritis, and 1 had bilateral pneumonia. Samples were taken from lungs and injected into embryonating eggs, which died in <2 days. The isolated virus was denominated A/crested eagle/Belgium/01/2004. The antigenic subtyping as H5N1 was made by hemagglutination inhibition using 12 monospecific polysera. The diagnosis was confirmed by nucleoprotein gene (general for type A influenza) and H5-specific reverse transcriptase–polymerase chain reaction (RT-PCR) (primers summarized in Table). Sample quality was controlled by using 18S rRNA as housekeeping control gene with bird-conserved primers (Table), validated against a variety of bird species (including chicken, duck, goose, and crow) and tissues (spleen, brain, lung, trachea, cecum, liver; unpub. data). The high pathogenicity of the virus was confirmed by measuring the intravenous pathogenicity index in chickens (IVPI = 2.94).

**Table:** Primers used for human (h) and avian (a) diagnosis, subtyping, and sequencing

Primer	Specificity	Host	Primer sequence (5´–3´)	PCR product (bp)	Reference*
NP1200F	Flu A	h, a	CAGRTACTGGGCHATAAGRAC	330	(6)
NP1529R	Flu A	h, a	GCATTGTCTCCGAAGAAATAAG		(6)
NP 1222 F	Flu A	h	CAGRAGYGGAGGRAAYACYAAYC	245	This study (IT)
NP 1467 R	Flu A	h	CGGGYTCGYTGCCTTYTCGTC		This study (IT)
BHAA	Flu B	h	GTGACTGGTGTGATACCACT	900	(7)
BHAD2	Flu B	h	TGTTTTCACCATATTGGGGC		(7)
BHAB	Flu B	h	CATTTTGCAAATCTCAAAGG	767	(7)
BHAC2	Flu B	h	TGGAGGCAATCTGCTTCACC		(7)
H5-155F	Flu A/H5	h, a	ACACATGCYCARGACATACT	545	(6)
H5-699R	Flu A/H5	h, a	CTYTGRTTYAGTGTTGATGT		(6)
H5 1 F	Flu A/H5	h	GCCATTCCACAACATACACCC	401	Mod. from (8)
H5 2 R	Flu A/H5	h	TTAATTCTCTATCCTCCTTTCCAA		Mod. from (8)
H5 IS 1F	Flu A/H5	h	CTTGCGACTGGGCTCAGAAAT	645	This study (IT)
H5 IS 2R	Flu A/H5	h	CCTTCCAACGGCCTCAAACTG		This study (IT)
β act 5	βactine	h	AACACCCCAGCCATGTAC	181	This study (IT)
β act 6	βactine	h	GTAGTCAGTCAGGTCCCG		This study (IT)
β act 7	βactine	h	AACACCCCAGCCATGTAC	144	This study (IT)
β act 8	βactine	h	GCCAGCCAGGTCCAGACG		This study (IT)
18S-F908	18S	a	AGCGAAAGCATTTGCCAAGA	401	This study (SVB)
18S-R1309	18S	a	AGTCTCGTTCGTTATCGGAATT		This study (SVB)
H5-614F	HA sequencing	a	GARGAYCTTYTRRTAHTRTGG	645	This study (MB)
H5-1259R	HA sequencing	a	CYTCRAAHTGRGTGTTCATT		This study (MB)

*IT, Isabelle Thomas; SVB, Steven Van Borm; MB, Marc Boschmans; Mod., modeled.

After tracing birds that had passed through the customs inspection center during the at-risk period, the Federal Agency for Food Chain Safety killed several batches of birds, notably 2 parrots at the customs inspection center, 200 parrots in a quarantine center, and 450 birds in another quarantine center. They were tested by RT-PCR and virus isolation on embryonating egg or cell culture. All were negative for the H5N1 strain. As soon as the results from the tested hawk-eagles were known, 25 persons who had been in direct or indirect contact with the infected eagles in Brussels airport were rapidly traced back, examined, and given oseltamivir prophylaxis. The Thai man and a close contact went to the police immediately after hearing about the diagnosis of avian influenza in the news. They were immediately brought to Antwep University Hospital, put into an isolation unit for 4 days, monitored, and given oseltamivir prophylaxis. All exposed persons remained asymptomatic, except for the veterinarian who euthanized the birds; bilateral conjunctivitis developed 2 days after he examined the birds. His family was given prophylaxis as well.

The Scientific Institute of Public Health informed the health authorities of all EU member states, as well as the appropriate EU authorities. Passengers who had taken the same flights as the bird smuggler were informed by the media to seek medical advice if they had any influenzalike symptoms within 7 days after the flight. Swabs (2 nasal and 1 throat) from 23 persons (21 custom officers, the smuggler, and a close contact) as well as a tear swab collected from the veterinarian, who had conjunctivitis, were tested by using nested RT-PCR to achieve maximal sensitivity: one to detect influenza A and B and the second for subtyping of influenza A H5 (Table). Primers from the human β-actin gene were used as housekeeping genes for control and designed by using a published sequence (GenBank accession no. M10277) (Table). All samples (including the tear swab) were negative for influenza A or B and confirmed negative for H5. The conjunctivitis tear swab sample was confirmed to be negative by injection into embryonating eggs.

**Figure 2:**
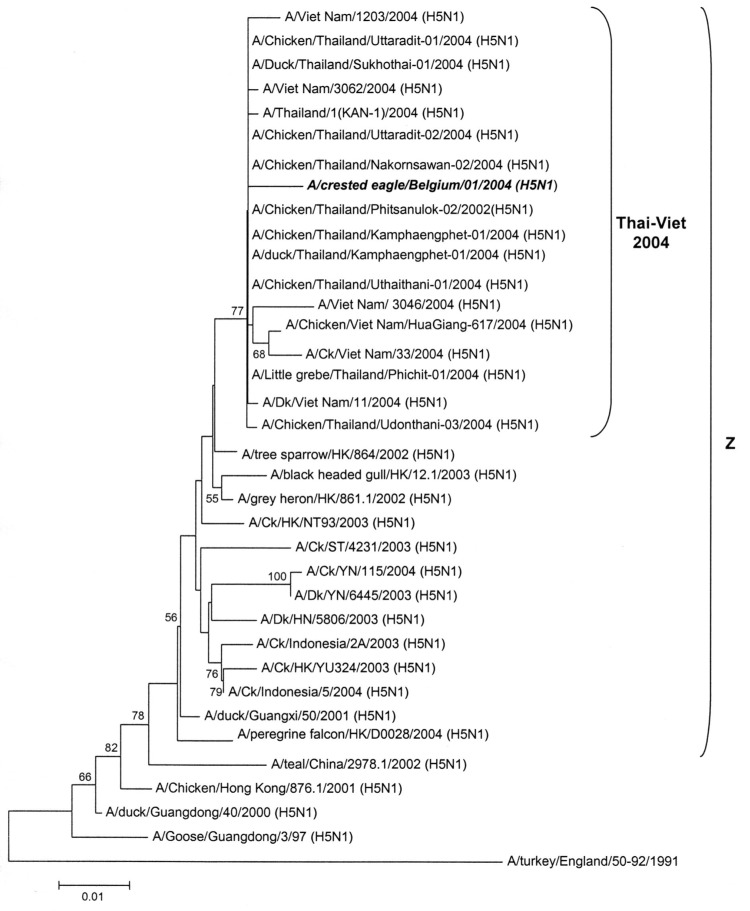
Neighbor-joining phylogenetic tree (rooted to A/turkey/England/50-92/1991) based on the alignment of a 654-bp fragment of the hemagglutinin gene of A/crested eagle/Belgium/2004 (***bold italic***), including the cleaving site. Bootstrap values >50 (1,000 replicates) are indicated near the branches. The Z cluster refers to Li et al. (*10*).

A 645-bp part of the hemagglutinin (HA) gene, spanning the HA cleavage site, was sequenced 3 times from each of 3 independent RT-PCR amplification products (primers specified in Table). The amplicons were cloned into a pCR2.1-TOPO vector (TOPO TA cloning kit, Invitrogen, Carlsbad, CA, USA) and plasmid DNA from positive colonies was purified (Qiaprep miniprep kit, Qiagen, Valencia, CA, USA) before sequencing (BigDyeTerminator v.3.1 cycle sequencing kit, Applied Biosystems, Foster City, CA, USA). The 645-bp partial HA sequence (GenBank accession no. AY861372) of A/crested eagle/Belgium/01/2004 contained 5 mutations relative to its nearest isolate A/Ck/Thailand/9.1/2004 (H5N1) (GenBank accession no. AY651328; identity score 0.992), including 1 change in the HA cleavage site, resulting in a unique arginine (R) > lysine (K) replacement, which contains 6 basic amino acid residues KRRKKR, whereas all Thai isolates from 2003 and 2004 have RRRKKR (6-8). As the occurrence of multiple basic amino acids in the cleavage site marks highly pathogenic H5 and H7 influenza A strains (9), this finding confirms the results of the IVPI experiment. We aligned the partial HA sequence to 35 representative GenBank entries (ClustalW multiple alignment) and calculated a neighbor-joining phylogenetic tree based on this alignment (Figure 2 [10]). High bootstrap values support the clustering of A/crested eagle/Belgium/01/2004 together with strains from the current Asian H5N1 epidemic. Moreover, all of these 2004 Thai-Viet strains clearly belong to the previously described highly pathogenic Z genotype cluster (10).

## Conclusions

The simultaneous resurgence of the H5N1 epidemic across several countries during the summer of 2004 indicated that the virus has become endemic in Asian poultry. This situation represents an increasing threat for public health since constant multiplication and circulation of the virus might help selecting mutations that adapt the virus to new hosts. This continuous evolution (10) is supported by our study, which showed that, although the A/crested eagle/Belgium/01/2004 virus still belongs to the Z genotype, an amino acid change was observed in the HA cleavage site. The exchange of an arginine by a lysine conserved 6 basic amino acids in the cleavage site, a molecular structure demonstrated by reverse genetics studies as being the optimal sequence for highly pathogenic avian influenza virus cleavability and pathogenicity (11).

Although *Spizaetus nipalensis*, a CITES2-listed species (Convention on International Trade in Endangered Species), is well distributed in the H5N1 affected regions in Thailand (12), no details are currently available that might explain how the birds got infected. One possibility is that they were fed infected chicken carcasses before their departure to Europe. The birds may thus have been infected very shortly before their transportation. This scenario might explain why no clinical symptoms were found. Alternatively, avian wildlife may have a higher resistance to the disease before they exhibit clinical signs and then die suddenly. Preliminary quantitative real-time RT-PCR data collected in our laboratory (data not shown) support this explanation as they show a viral load in the eagles similar to poultry infected with highly pathogenic avian influenza (13). In a zoo in Cambodia, a variety of free-flying and captive birds, including raptors, were reported as being infected with the H5N1 virus (available from http://www.fao.org/docs/eims/upload/159535/AVIbull016.pdf). The disease appeared first in raptors, including hawk-eagles, in the first 2 to 3 days, indicating their high susceptibility. They were most probably fed infected chicken carcasses. The only other report of H5N1 in wild raptors consists of a single peregrine falcon found dead in Hong Kong (available from http://www.oie.int/eng/info/hebdo/AIS_60.htm). Unfortunately, no histopathologic confirmation of the cause of death or viral load assessment studies about this case have been communicated. There have also been 2 reports of avian influenza infections of falcons with H7 highly pathogenic avian influenza virus. Manvell et al. (14) reported the isolation of a highly pathogenic avian influenza virus of H7N3 subtype from a peregrine falcon dying in the United Arab Emirates. During the highly pathogenic avian influenza virus outbreaks in Italy in 2000, an H7N1 virus was isolated from a Saker Falcon that died 3 days after normal hunting activity (15). The raptor had a sudden onset of depression, weakness, and anorexia the day after normal hunting activity and died 2 days later without further clinical signs.

This study demonstrates that international air travel and smuggling represent major threats for introducing and disseminating H5N1 virus worldwide. Hunting with falcons is practiced in several countries around the world. Here, the falconer who ordered the birds already owned 4 other eagles of the same species. The 2 birds detected by customs may reflect a much larger underlying problem of bird smuggling. Such birds easily remain undetected because customs officers are essentially focused on metal objects, although airport scanners can theoretically detect bones of animals. Specific methods for systematically detecting live animals (e.g., trained dogs) should be considered at airports and borders (5). This method is now under evaluation in Belgium.
